# Gene Therapy and Epigenetic Modulation in Chronic Pain: A Future Without Opioids?

**DOI:** 10.7759/cureus.94508

**Published:** 2025-10-13

**Authors:** Courage O Idahor, Miriam A Okorie, Sarah Mokobia, Prize Erhuanga, Ndidiamaka Ogbonna, Olamide Ogunfuwa, Ovie M Etoroma, Itua J Abhulimen, Ekene Chinedu

**Affiliations:** 1 Emergency Medicine, Nottingham University Hospital NHS Trust, Nottingham, GBR; 2 Medicine and Surgery, Ebonyi State University, Abakiliki, NGA; 3 Emergency Medicine, James Paget University Hospitals NHS Foundation Trust, Great Yarmouth, GBR; 4 Medicine, University of Benin, Benin, NGA; 5 Family Medicine, Edo State University Uzairue, Uzairue, NGA; 6 Emergency Medicine, Sandwell and West Birmingham NHS foundation Trust, Birmingham, GBR; 7 Faculty of Pharmacy, University of Benin, Benin, NGA; 8 Family Medicine, University of Benin, Benin, NGA; 9 General Medicine, NHS Lanarkshire, Bothwell, GBR

**Keywords:** chronic pain, epigenetic modulations, epigenetic mutations, gene therapy, opioids

## Abstract

Chronic pain represents a pervasive and complex clinical challenge that continues to affect millions worldwide, often resulting in significant personal suffering, disability, and socioeconomic burden. Despite advances in pharmacologic and interventional approaches, management remains heavily reliant on opioids, contributing to widespread dependence, tolerance, and the ongoing global opioid crisis. These challenges underscore the urgent need for safer, more sustainable therapeutic alternatives that target the biological roots of pain rather than merely alleviating symptoms. This narrative review explores gene therapy and epigenetic modulation as transformative approaches capable of reshaping chronic pain management.

Gene therapy enables precise manipulation of pain-related genes through viral and non-viral delivery systems, offering the potential to modify ion channels, neurotransmitter receptors, and inflammatory mediators implicated in neuropathic pain. Concurrently, epigenetic modulation through mechanisms such as DNA methylation, histone modification, and non-coding RNA regulation offers a reversible means of reprogramming aberrant gene expression patterns that sustain chronic pain states. The synergistic integration of these two modalities holds promise for durable, personalized, and opioid-independent pain control.

While promising, these strategies face challenges including delivery precision, ethical and safety concerns, cost, and regulatory barriers. Continued interdisciplinary research involving molecular biologists, pain specialists, and clinical scientists is essential to ensure safe translation into practice. Harnessing the power of gene and epigenetic therapies may redefine the future of pain medicine, paving the way for long-term relief without opioid dependence.

## Introduction and background

Pain was traditionally conceptualised as a direct result of tissue damage, following a biomedical model that assumed a one-to-one relationship between physical injury and pain symptoms [1,2]. In cases where no organic pathology could be found, psychological explanations were often suggested, which contributed to the stigmatisation of patients reporting persistent pain without clear physical causes [3,4]. This linear framework proved inadequate, especially in the context of chronic pain, where the subjective nature of pain and its persistence beyond tissue healing could not be explained solely by anatomical or physiological abnormalities [5,6]. In response, the biopsychosocial model was introduced, first articulated by George Engel and later expanded by Fordyce and Loeser, emphasising the dynamic interplay between biological, psychological, and social influences in pain experience [7-11]. This model has reshaped clinical understanding of chronic pain and has encouraged the development of more nuanced, patient-centred approaches to treatment [12].

Chronic pain, defined as pain lasting longer than three months, is a global health burden with an estimated prevalence of 20% in the general population [13,14]. Neuropathic pain, a common subtype, is caused by lesions or dysfunction in the somatosensory nervous system and is characterised by spontaneous pain, hyperalgesia, and allodynia [15,16]. The impact of chronic pain extends beyond physical discomfort, affecting emotional well-being, functional capacity, and quality of life. Many patients experience anxiety, depression, sleep disturbances, and social isolation as secondary consequences of long-term pain [17-19]. The burden is also economic, with chronic pain contributing to increased healthcare utilisation, reduced work productivity, and long-term disability [20].

Pharmacologic management remains a cornerstone of chronic pain therapy, with opioids historically occupying a central role [21]. The widespread adoption of opioids in the 1990s for non-cancer pain was driven by advocacy for better pain management and the misconception that long-term opioid use carried minimal risk for addiction [22]. However, the consequences of this shift became evident in the early 2000s as rates of opioid misuse, dependence, and overdose escalated [23]. Data from the National Survey on Drug Use and Health showed that first-time non-medical use of prescription opioids rose from 628,000 in 1990 to over 2.4 million in 2004 [14]. During the same period, treatment admissions for opioid abuse increased by more than 180% and emergency visits related to opioid misuse spiked by 45%. The recognition of these risks has led to stricter prescribing regulations and renewed interest in non-opioid and non-pharmacologic alternatives for pain management [24,25].

This shift has opened a path for innovative biological therapies that target the molecular mechanisms underlying chronic pain. Among these, gene therapy and epigenetic modulation have emerged as promising strategies that offer the potential for sustained pain relief by altering gene expression patterns within the nervous system [17,19,20]. Chronic pain is associated with long-term changes in neuronal function that result from altered gene activity in the spinal cord and brain [18,21]. These changes contribute to central sensitisation, where neurons become hyperresponsive, amplifying pain signals even in the absence of ongoing tissue damage.

Epigenetic mechanisms, which influence gene expression without changing the DNA sequence, are believed to play a pivotal role in these processes. These mechanisms include DNA methylation, histone modification, and regulation by non-coding RNAs such as microRNAs [22,23]. Epigenetic changes are dynamic and can be induced by environmental stimuli, inflammation, stress, or injury, allowing the nervous system to adapt at the molecular level. While this plasticity is essential for processes like learning and memory, it can also lead to maladaptive states that support the persistence of pain [24].

Preclinical studies have shown how specific epigenetic changes in the spinal cord are linked to pain hypersensitivity. For example, animal models of inflammatory pain using complete Freund’s adjuvant (CFA) have demonstrated phosphorylation of MeCP2, a transcriptional repressor, in neurons of the dorsal horn. This modification is associated with the repression of genes involved in nociceptive signalling and a reduction in pain thresholds [20,21]. Similarly, formalin-induced pain in mice has been shown to increase MeCP2 mRNA levels, indicating that transcriptional regulation plays a central role in pain modulation [22].

Neuropathic pain models provide further evidence for the role of histone modifications in chronic pain. In a partial sciatic nerve ligation model, increased expression of MCP-3, a pro-inflammatory chemokine, was observed in spinal astrocytes. This was linked to a decrease in the repressive histone mark H3K27me3 at the MCP-3 promoter region [23]. Notably, administration of an anti-MCP-3 antibody reversed pain behaviours in the animals, and these effects were dependent on interleukin-6 (IL-6), as MCP-3 expression was abolished in IL-6 knockout mice. These findings suggest that inflammatory signalling and epigenetic deregulation interact to sustain chronic pain [24].

Gene therapy presents another promising avenue by enabling the direct delivery of genes that encode analgesic proteins, neuroprotective factors, or inhibitory neurotransmitters [22,25]. Various viral vectors have been used to introduce therapeutic genes into targeted tissues, particularly the spinal cord and dorsal root ganglia. For instance, gene transfer of glutamic acid decarboxylase (GAD) enhances gamma-aminobutyric acid (GABA) production and has been shown to reduce neuropathic pain in preclinical models [21]. Another approach involves the delivery of siRNA to silence genes such as Nav1.7, a voltage-gated sodium channel that plays a critical role in pain transmission. Knockdown of Nav1.7 expression has demonstrated significant analgesic effects in rodent models of neuropathic pain [24].

The integration of gene and epigenetic therapy holds great promise for transforming chronic pain treatment. These modalities offer the potential for long-lasting relief without the drawbacks of opioids or repeated pharmacologic dosing [19,22]. However, their translation into clinical practice is not without challenges. Concerns regarding vector safety, immune responses, off-target effects, and ethical considerations must be carefully addressed [23]. Moreover, large-scale clinical trials are needed to validate efficacy and establish regulatory pathways for approval [24,25].

Despite these hurdles, the momentum behind gene-based pain therapies continues to build. As the molecular understanding of chronic pain deepens, the integration of epigenetic and genetic interventions could redefine pain management in the future. This shift toward precision medicine aims not just to suppress symptoms but to address the root mechanisms that sustain chronic pain. In doing so, it offers a hopeful prospect: a future where effective pain relief is possible without reliance on opioids [25].

## Review

The role of gene therapy in chronic pain

Gene therapy represents a rapidly evolving frontier in the management of chronic pain, offering new hope for conditions that have historically been refractory to conventional treatments. Chronic pain, especially of neuropathic origin, is often sustained by persistent changes in gene expression within the nervous system. By intervening at the genetic level, gene therapy aims to modulate the fundamental mechanisms responsible for pain transmission and maintenance, moving beyond symptomatic relief toward disease modification [24]. The concept of gene therapy broadly involves the introduction, removal, or alteration of genetic material within a patient’s cells with the intention of treating or preventing disease. This can be achieved through a variety of delivery approaches, most commonly categorised as viral or non-viral systems [26].

Viral vectors are the mainstay of gene delivery in research and emerging clinical applications. These vectors, including adeno-associated viruses (AAV), lentiviruses, and herpes simplex viruses (HSV), are engineered to efficiently deliver therapeutic genes into target cells without causing pathogenic infection [27]. Each viral system has distinct advantages. For instance, AAV vectors are favoured for their low immunogenicity and sustained gene expression. In contrast, HSV vectors exhibit strong tropism for sensory neurons, making them highly suitable for pain modulation strategies [28]. Lentiviral vectors enable stable integration of therapeutic genes into the host genome, facilitating long-term gene expression. However, concerns about insertional mutagenesis and potential oncogenesis have prompted the development of safer, non-integrating systems [29]. In contrast, non-viral delivery methods, such as liposomes, nanoparticles, and naked plasmid DNA, offer improved safety profiles and ease of manufacture but are generally less efficient in achieving durable gene transfer, particularly in neuronal tissues [30]. A balance between efficacy, safety, and the biological characteristics of the target tissue thus determines the choice of vector system.

Central to the promise of gene therapy in chronic pain is the identification and targeting of molecular drivers of nociception. The pain pathway is governed by a complex interplay of ion channels, neurotransmitter receptors, inflammatory mediators, and intracellular signalling cascades. Genetic interventions have focused on modulating the expression or function of key components within these pathways to disrupt the transmission or amplification of pain signals [31]. Among the well-characterised targets are voltage-gated sodium channels, particularly NaV1.7, NaV1.8, and NaV1.9, which are predominantly expressed in peripheral sensory neurons and play critical roles in action potential generation and propagation [32]. Mutations in the SCN9A gene, which encodes NaV1.7, are linked to rare inherited pain disorders, ranging from congenital insensitivity to pain to paroxysmal extreme pain disorder, highlighting its importance in human nociception [33]. The feasibility of modulating NaV1.7 expression through gene therapy has therefore been a significant area of investigation.

Preclinical studies have utilised viral vectors to deliver small interfering RNAs (siRNAs) or short hairpin RNAs (shRNAs) targeting NaV1.7, effectively silencing its expression in dorsal root ganglion (DRG) neurons. These approaches have resulted in significant attenuation of mechanical and thermal hyperalgesia in rodent models of neuropathic pain, with some effects lasting for several weeks following a single administration [34]. Similarly, gene therapy has been used to regulate other sodium channels such as NaV1.8, as well as calcium channels and potassium channels, each with demonstrated efficacy in altering pain thresholds in experimental models [35]. Beyond ion channels, other promising targets include transient receptor potential (TRP) channels, such as TRPV1, which are involved in the transduction of noxious heat and inflammatory stimuli. AAV-mediated delivery of dominant-negative TRPV1 constructs has been shown to reduce heat hyperalgesia in animal models of inflammation and nerve injury [36].

Gene therapy strategies have also focused on neurotransmitter systems. The gamma-aminobutyric acid (GABA)-ergic system is a principal inhibitory network within the spinal cord that regulates pain signalling. Diminished GABAergic tone is a hallmark of many chronic pain states. Several studies have used HSV or AAV vectors to deliver the gene encoding GAD, the enzyme responsible for GABA synthesis, directly into the DRG or spinal cord. This approach has led to increased GABA production at pain-relevant sites, resulting in sustained pain relief in models of neuropathic and inflammatory pain [36]. In a notable early-phase clinical trial, HSV-mediated GAD gene transfer produced measurable reductions in pain intensity among patients with intractable cancer pain, demonstrating the translational potential of these findings [22]. Gene therapy has also been used to enhance endogenous opioid pathways. For example, delivery of proenkephalin, a precursor of endogenous enkephalins, via viral vectors has shown efficacy in producing analgesia without the side effects associated with systemic opioid administration [37].

The inflammatory microenvironment surrounding injured nerves and central pain circuits presents additional targets for genetic modulation. Cytokines such as IL-6, tumour necrosis factor-alpha (TNF-alpha), and monocyte chemoattractant protein-1 (MCP-1) are upregulated in neuropathic and inflammatory pain states, perpetuating neuronal sensitisation and glial activation. Gene silencing techniques or gene delivery of anti-inflammatory cytokines, such as interleukin-10 (IL-10), have successfully reduced pain behaviours in animal models [38]. One notable approach involved AAV-mediated delivery of IL-10 to the spinal cord, resulting in suppression of glial activation and reversal of established neuropathic pain [39]. Conversely, strategies that block chemokine signalling, such as knockdown of MCP-1 or its receptor CCR2, have also produced promising results in preclinical studies [40].

Clinical translation of gene therapy for chronic pain has progressed steadily but remains cautious due to the complexities of nervous system targeting and safety considerations. Early-phase clinical trials have primarily explored HSV-based vectors for delivery to sensory ganglia or peripheral nerves, exploiting the natural neurotropism of HSV to minimise off-target effects. In a phase I clinical trial, HSV vector-mediated delivery of the human preproenkephalin gene to patients with cancer-related pain was well-tolerated and produced reductions in pain scores for several weeks after a single injection, without significant adverse effects [22]. Subsequent studies have replicated these results, supporting the safety and feasibility of localised gene therapy in humans [41]. However, challenges persist in achieving precise control over gene expression, optimising vector dosing, and mitigating immune responses to the vector or transgene product [42]. The possibility of long-term gene integration and off-target effects remains a focus of ongoing preclinical and clinical investigations.

Durability of pain relief is a major advantage of gene therapy over conventional pharmacotherapy. Traditional drugs for chronic pain often require repeated dosing, with associated risks of tolerance, dependence, and side effects. In contrast, gene therapy offers the potential for persistent modulation of pain pathways following a single administration, with effects lasting weeks to months or even longer, depending on the vector and target [43]. Long-term studies in animal models have demonstrated stable transgene expression and sustained analgesia, although some interventions have shown diminishing effects over time, underscoring the need for continued research into gene silencing and vector optimisation [44]. Importantly, the ability to reversibly regulate gene expression, for example, through inducible promoter systems or re-administration of vectors, could allow for even greater flexibility and safety in future clinical protocols [45].

Despite these promising advances, significant hurdles remain before gene therapy can be widely adopted in the clinic for chronic pain. Issues of safety, scalability, regulatory approval, and ethical concerns regarding genetic manipulation in non-life-threatening conditions must be addressed. The balance between efficacy and risk is particularly delicate given the diversity of chronic pain etiologies and patient populations [46]. Nonetheless, the field is moving forward rapidly, propelled by advances in vector technology, genome editing tools such as CRISPR/Cas9, and a growing understanding of pain neurobiology [47]. Ongoing trials are investigating combinations of gene therapy with other modalities, such as cell therapy or neuromodulation, to further enhance efficacy and durability [48].

Gene therapy’s capacity to offer durable, mechanism-based pain relief positions it as a transformative strategy in the pain field. By targeting the underlying genetic and molecular drivers of chronic pain, these interventions could reduce or even eliminate the need for continuous pharmacologic therapy, improve patient quality of life, and address the societal costs associated with pain-related disability and opioid use [49]. As research continues to advance, there is genuine optimism that gene therapy will transition from a research tool to a standard clinical option for select chronic pain syndromes. This therapeutic approach would fundamentally transform the philosophy of pain management by prioritising precise and lasting alteration of the underlying disease mechanisms, rather than merely suppressing symptoms [50].

Epigenetic modulation in chronic pain

Epigenetics has become a crucial area of investigation in the quest to understand and treat chronic pain, revealing layers of complexity in gene regulation that extend far beyond the static information encoded in DNA [51]. At its core, epigenetics refers to heritable changes in gene expression that occur without altering the underlying DNA sequence, instead relying on chemical modifications to DNA or its associated proteins, as well as the regulatory effects of non-coding RNAs [52]. These mechanisms allow the genome to respond dynamically to environmental cues and cellular conditions, orchestrating context-specific gene activity throughout development and adult life [53]. The major epigenetic processes include DNA methylation, histone modification, and regulation by non-coding RNAs, all of which have been implicated in both the development and persistence of chronic pain syndromes [17].

DNA methylation is one of the most extensively studied epigenetic modifications. It typically involves the addition of a methyl group to the 5’ position of cytosine rings, usually at CpG dinucleotides, catalysed by DNA methyltransferases (DNMTs) [54]. In promoter regions, increased DNA methylation is usually associated with transcriptional repression, while demethylation can facilitate gene activation [55]. Histone modifications represent another major category, encompassing acetylation, methylation, phosphorylation, ubiquitination, and sumoylation of histone proteins around which DNA is wrapped [56]. These modifications alter chromatin structure and influence the accessibility of transcriptional machinery to genomic DNA [57]. For instance, histone acetylation-mediated by histone acetyltransferases (HATs), typically relaxes chromatin and promotes gene expression, whereas deacetylation by histone deacetylases (HDACs) condenses chromatin and suppresses transcription [58]. Beyond these, histone methylation at specific lysine residues can either activate or repress transcription depending on the context, as seen with H3K9 or H3K27 trimethylation in pain-related genes [59,60]. Non-coding RNAs, including microRNAs (miRNAs), long non-coding RNAs (lncRNAs), and circular RNAs, serve as post-transcriptional regulators of gene expression and can also modulate epigenetic states by interacting with chromatin-modifying complexes [61].

One of the most compelling features of epigenetic regulation is its responsiveness to environmental stimuli. Chronic pain does not arise solely from a static injury or a fixed genetic background; rather, it emerges through the interplay of genetics, experience, and environmental factors, all of which converge on the epigenome [62]. Physical injury, ongoing inflammation, psychological stress, and even lifestyle factors such as diet and physical activity can influence epigenetic marks in the nervous system [63,64]. For example, exposure to persistent inflammatory cytokines after nerve injury has been shown to alter DNA methylation and histone modification patterns in DRG neurons and spinal cord glia, leading to stable changes in the transcription of pain-related genes [65,66]. Stressful experiences, including early-life adversity, have also been demonstrated to induce lasting epigenetic changes that sensitise individuals to chronic pain in adulthood [67]. These findings support a model in which the epigenome serves as a molecular memory of both acute and chronic insults, encoding a persistent vulnerability to pain [68].

At the molecular level, epigenetic mechanisms modulate a wide array of genes and pathways implicated in nociception and pain chronification. NMDA receptors, central to synaptic plasticity and central sensitisation in pain, are one such target [69]. Upregulation of NMDA receptor subunit genes has been linked to reduced DNA methylation and increased histone acetylation at their promoters following nerve injury [70]. Similarly, genes encoding voltage-gated sodium channels (such as NaV1.7 and NaV1.8), which are critical for action potential generation in sensory neurons, can be epigenetically upregulated, contributing to neuronal hyperexcitability and pain hypersensitivity [71]. Increased expression of these channels has been traced to histone acetylation and DNA demethylation at promoter sites [72]. The endogenous opioid system, which modulates pain inhibition, is also under epigenetic control. In animal models, chronic exposure to opioids or persistent pain has been associated with increased methylation of the mu-opioid receptor (MOR) gene promoter, resulting in reduced receptor expression and diminished opioid responsiveness [73]. This may partially explain the clinical challenge of opioid tolerance in chronic pain management [19].

The role of histone modification in pain states is equally significant. After peripheral nerve injury, increased expression and activity of HDACs in the spinal cord have been observed, leading to decreased acetylation of histones associated with genes that normally dampen pain signalling [74]. Pharmacologic inhibition of HDACs has been shown to reverse this effect, restoring the expression of pain-inhibiting genes and attenuating hypersensitivity in preclinical models [70]. In parallel, pro-inflammatory genes such as those encoding cytokines (e.g., TNF-alpha, IL-6) and chemokines (e.g., MCP-1/CCL2) often display decreased levels of repressive histone marks like H3K27 trimethylation after nerve injury, contributing to sustained neuroinflammation and central sensitisation [75]. H3K9 and H3K27 methylation also influence the expression of genes like BDNF, which plays a role in pain plasticity [20]. The regulatory actions of non-coding RNAs further add to this complexity. Specific miRNAs, including miR-124, miR-155, and miR-21, have been shown to target mRNAs encoding pro- or anti-nociceptive factors, thus shaping the transcriptomic landscape of pain pathways [76]. For example, downregulation of miR-124 has been observed in neuropathic pain models and is associated with microglial activation and increased production of inflammatory mediators in the spinal cord [77]. Long non-coding RNAs, such as Kcna2 antisense RNA, can suppress potassium channel gene expression, contributing to neuronal hyperexcitability [78].

Beyond neuronal populations, glial cells such as microglia and astrocytes are subject to epigenetic regulation, and their activation is a hallmark of chronic pain [79]. DNA methylation changes in microglial cells can alter the expression of genes involved in cytokine production and phagocytic activity [80]. Epigenetic priming of glia can result in a state of “trained immunity,” where glial cells display exaggerated responses to subsequent insults, thereby perpetuating pain [81]. This phenomenon has direct implications for the maintenance and recurrence of chronic pain states following a range of peripheral injuries or inflammatory conditions [82].

The potential to therapeutically modulate epigenetic mechanisms has generated great interest as a novel approach to pain management. Among the most actively studied pharmacologic interventions are histone deacetylase inhibitors (HDACis), which block the removal of acetyl groups from histones, leading to a more relaxed chromatin state and upregulation of gene expression [83]. HDACis such as suberoylanilide hydroxamic acid (SAHA), trichostatin A (TSA), and valproic acid have demonstrated efficacy in reducing neuropathic and inflammatory pain behaviours in rodent models [84]. These agents appear to restore the expression of GABAergic and other inhibitory genes while also suppressing pro-inflammatory gene transcription [85]. Notably, HDAC inhibition has been shown to block microglial activation and reduce the production of cytokines implicated in central sensitisation [86]. The specificity of action depends on the particular HDAC isoforms targeted, suggesting that more selective HDACis may offer pain relief with fewer side effects [87].

DNA methyltransferase inhibitors (DNMTis), such as 5-azacytidine and RG108, have also been evaluated for their analgesic potential [88]. By blocking the activity of DNMTs, these compounds reduce DNA methylation at specific gene promoters, thereby promoting transcription of genes that suppress pain transmission or neuroinflammation [89]. For instance, 5-azacytidine administration in models of nerve injury has resulted in decreased pain behaviours, partly through demethylation and reactivation of the MOR gene and other inhibitory pathways [70]. Although the clinical application of DNMTis in pain remains largely experimental, their success in preclinical studies highlights the feasibility of targeting the pain epigenome therapeutically [88].

Therapies based on non-coding RNAs are another frontier. Efforts to modulate miRNA activity have involved either supplementing downregulated miRNAs (using miRNA mimics) or inhibiting overexpressed miRNAs (using antagomirs) [90]. For example, administration of miR-124 mimics in animal models has attenuated microglial activation and reduced neuropathic pain [91]. Conversely, inhibiting miR-21 - a miRNA upregulated in nerve injury and associated with neuroinflammation -has produced significant analgesia in rodent studies [76]. Long non-coding RNAs are similarly being explored as potential targets, given their ability to scaffold chromatin modifiers and shape gene expression over broad genomic domains [92].

A growing body of clinical and translational evidence supports the importance of epigenetic mechanisms in human pain syndromes. Postmortem analyses of human DRG and spinal cord tissue from chronic pain patients have revealed altered DNA methylation and histone modification patterns at key pain-related genes [19]. Peripheral blood samples from patients with fibromyalgia, chronic back pain, and complex regional pain syndrome (CRPS) have shown changes in miRNA expression profiles that correlate with pain intensity and duration [93]. Pilot studies investigating the use of valproic acid, an HDACi, in neuropathic pain patients have reported modest but significant reductions in pain scores, providing early clinical validation for this approach [94]. Larger, randomised trials are needed to further establish efficacy and safety, particularly given concerns about the broad impact of epigenetic drugs on other physiological systems [95].

Ongoing research into epigenetic modulation is also intersecting with other emerging technologies, including gene editing and personalised medicine. The advent of CRISPR-based epigenome editing now permits highly targeted modification of specific regulatory elements, offering the possibility of correcting pathogenic epigenetic marks in a cell- and gene-specific manner [96]. In the future, integration of epigenetic biomarkers with genetic and clinical data may enable the development of precision pain therapeutics tailored to individual epigenomic profiles [97]. There is also an active investigation into the three-dimensional chromatin architecture in neurons and how higher-order chromatin looping may influence pain gene expression [98].

Despite the promise, several challenges must be addressed before epigenetic therapies can be widely adopted for chronic pain. Off-target effects, the risk of unintended global changes in gene expression, and incomplete understanding of the long-term consequences of epigenetic drugs remain important considerations [99]. Additionally, the reversibility and dynamic nature of epigenetic marks mean that ongoing environmental exposures could potentially undermine therapeutic gains unless underlying triggers are also addressed [100]. Nevertheless, the convergence of molecular biology, neuroscience, and pharmacology in the field of pain epigenetics is creating new avenues for durable and mechanism-based pain relief, potentially transforming how clinicians approach chronic pain in the coming decades [53]. As the field continues to evolve, there is cautious optimism that epigenetic modulation, whether through small molecules, RNA-based strategies, or precision genome editing, will move from experimental models to mainstream clinical practice. Ultimately, by targeting the molecular switches that govern pain signalling and memory, epigenetic therapies may offer not just symptomatic relief but the possibility of true disease modification for individuals suffering from chronic pain [101].

Gene therapy and epigenetics synergy in pain management

Combining gene therapy with epigenetic modulation is gaining attention as a way to strengthen and extend pain relief, especially in cases of chronic pain where conventional pain medications like opioids present with severe adverse effects. An earlier research was conducted on 2721 participants with chronic pain on prescription opioid pain medication, to evaluate the effects of certain genes on pain perception. Pain perception was measured on a scale of 1 to 10. 1-2-3 represented low pain tolerance, 4-5-6 were scored as moderate pain perception, and 7-8-9 or 10 showed high pain tolerance. The researchers discovered that people who felt less pain were 33% more likely to have a specific variation of the DRD1 gene compared to those who felt more pain. For those with a moderate pain threshold, variations in the COMT and OPRK genes showed up 25% and 19% more often, respectively, than in people with high pain sensitivity. Meanwhile, the DRD2 gene variant appeared 25% more frequently in individuals who reported higher pain levels than in those with moderate pain perception [102].

Millions of patients worldwide suffer from chronic pain, and it affects every aspect of their lives. Traditional painkillers, like opioids, are very useful in pain management, but they also have side effects such as dependence, respiratory depression, and sedation [103]. For this reason, researchers are turning to something new and much more targeted: combining gene therapy with epigenetics to not just manage the pain, but to address it at its molecular roots, creating more effective, targeted, and sustainable solutions [104]. Chronic pain does not affect everyone the same way. Each person experiences pain differently due to a unique mix of biological, psychological, and social influences. This understanding is part of the reason treatment is moving away from traditional opioid medications to a more patient-centred management [17].

Gene therapy and epigenetics are two distinct approaches that complement one another. Gene therapy involves the delivery of specific genes to the body’s cells to modify the function of genes associated with pain pathways. This can help to directly modulate pain responses by either enhancing or inhibiting certain biological processes [22]. On the other hand, epigenetics involves modifications to the expression of genes without changing the underlying genetic code itself. This includes alterations in DNA methylation, histone modification, and non-coding RNA activity, all of which can influence the way genes involved in pain are expressed [105]. By combining these two approaches, researchers hope to not only target pain at the molecular level but also ensure that these interventions have long-lasting effects. Epigenetic modifications can help to maintain the therapeutic changes brought about by gene therapy, offering a more sustainable solution to chronic pain management [106]. This combined approach holds the potential to not only enhance pain relief but also reduce the reliance on opioids and other pharmacological treatments, which are associated with significant risks such as addiction, tolerance, and side effects [107].

Gene therapy offers an alternative and better way of treating chronic pain by modifying the genes responsible for transmitting pain signals. Instead of just blocking pain temporarily, it aims to fix the underlying cause. This often involves using viral vectors to deliver genes that encode natural pain-relievers like GABA, anti-inflammatory cytokines, or opioid-like peptides. These techniques have already shown positive results in both animal models and early clinical trials [22]. Advanced tools like CRISPR-Cas9 allow scientists to precisely edit pain-related genes-such as SCN9A, which is responsible for the NaV1.7 sodium channel. In some inherited pain syndromes, a single edit has the potential to offer lasting or even permanent relief [33]. Studies by Fink et al. have demonstrated the safe and targeted delivery of gene therapies using HSV vectors to the DRG, reducing pain with minimal side effects [108].

The difference between gene therapy and epigenetics is that while gene therapy works at the DNA level, epigenetics modifies how those genes are expressed-without changing the DNA itself. Epigenetic mechanisms like DNA methylation, histone modification, and non-coding RNAs are recognised as key drivers in how pain develops and persists [109]. In chronic pain, the DNA in nerve and glial cells becomes “reprogrammed,” with altered methylation and histone patterns that keep pain pathways active [70]. Drugs that reverse these changes-like HDAC inhibitors-have shown promise in reducing inflammation and nerve pain [110]. Research by Massart et al. has also found common epigenetic patterns between the brain and immune cells, offering a new way to tailor treatment based on a patient’s molecular profile [104].

A combination of the long-term potential of gene therapy with the precision control of epigenetics is believed to be the future of pain management. Gene therapy can lay the groundwork by introducing beneficial genes, while epigenetic drugs can then boost and sustain their effects, for example, by making the chromatin more accessible for gene expression [111]. Using both approaches together - such as delivering anti-inflammatory genes while administering HDAC inhibitors - has shown significantly improved outcomes in early studies [112]. Moreover, epigenetic profiling can help tailor gene therapy vectors to individual patients, improving both safety and efficiency [20]. By examining a patient’s genome and epigenome, clinicians can predict which will respond best to which therapies [113]. For instance, methylation patterns in genes like BDNF, COX-2, and OPRM1 may indicate how someone will respond to treatment [114]. Mutations in pain-related genes like TRPV1 or NaV1.7 can be targeted directly with CRISPR-based editing [115]. On top of that, pharmacogenomic data helps guide drug choices and dosages, avoiding side effects and maximising benefit [116].

The use of CRISPR-Cas9 allows for precise editing of genes like SCN9A (a gene strongly linked to pain perception). Mutations in this gene have been associated with rare disorders where individuals can not feel pain at all [117]. By targeting this gene, researchers aim to create targeted pain relief with fewer side effects compared to systemic treatment [19]. But still, the delivery and durability of these effects can vary. Then, epigenetic drugs like HDAC inhibitors can help maintain the expression of therapeutic genes delivered through vectors. They help to keep DNA in a more open, transcriptionally active state, which might prevent the body from silencing those therapeutic genes over time [103]. Some research shows that combining gene delivery, such as viral vectors carrying genes that produce anti-inflammatory or inhibitory proteins, with epigenetic drugs can also enhance and prolong pain relief [118]. For instance, certain pain-modulating genes can be silenced over time due to histone modifications, but epigenetic therapies may reverse that [119]. This dual strategy reinforces the original gene therapy, providing a synergistic effect and is thought to help prevent the relapse of pain symptoms by sustaining gene activity where it matters most. 

Integrating gene therapy with epigenetic modulation offers a promising alternative for managing chronic pain, and by focusing on reducing symptoms, it aims to reset or reprogram the underlying cause at the base level. While more clinical work is needed, the potential for personalised, long-term, and non-addictive pain relief is believed to be a possibility [120].

Potential advantages of gene therapy and epigenetics in chronic pain

One of the most important benefits of gene therapy and epigenetics is their potential for sustainable pain relief without the need for ongoing opioid use, unlike traditional analgesics such as opioids that are associated with dependence, tolerance, and adverse effects [103]. Gene and epigenetic therapies aim to induce long-lasting pain management by changing the expression of pain-related genes. For example, chronic pain has been linked to specific epigenetic changes such as altered DNA methylation patterns in the prefrontal cortex and immune cells [104]. Gene therapy makes use of gene editing technology like CRISPR or zinc finger proteins to target and silence pain-related genes such as NaV1.7, a sodium channel implicated in chronic pain signalling. Silencing this gene has been shown to produce durable analgesia in animal models without triggering opioid dependence [121]. Furthermore, advanced gene therapies use viral vectors like AAV or HSV to deliver genetic material directly to pain-specific sites, such as the DRG. This localised delivery helps reduce systemic side effects typically seen with oral or systemic pain medications [122].

By sequencing a patient's genome and analysing epigenetic markers, clinicians can identify specific molecular targets that drive their pain. Gene therapies can then be used to upregulate or silence these targets, for example, including ion channels (NaV1.7, TRPV1), neurotransmitter receptors (NMDA, GABA), or inflammatory mediators, giving rise to a more focused and patient-centred care. This means that if a patient exhibits heightened expression of a nociceptive ion channel due to an epigenetic change, targeted gene silencing could reduce their pain more effectively than using systemic medications [123]. Different individuals perceive pain differently and also have varying responses to pain medications depending on their genetic and epigenetic variations. For instance, polymorphisms in genes like COMT, OPRM1, or SCN9A can change pain sensitivity, drug metabolism, and the effectiveness of opioid medications. Additionally, epigenetic modifications, such as DNA methylation or histone acetylation, can influence the expression of pain-related genes over time, especially in response to environmental stressors or chronic inflammation [103]. This targeted approach improves therapeutic efficacy, as well as reducing the risk of side effects and ineffective treatment cycles. Patients benefit from interventions that are targeted to their specific pain pathways, leading to faster relief, fewer adverse reactions, and a higher overall quality of life [44].

Chronic pain often goes deeper than the physical symptoms. The root cause is often due to changes in how genes are expressed in the nervous system. While traditional Pain medications usually mask the pain, gene therapy and epigenetic reprogramming aim to correct the source of the problem. Certain genes, like TRPA1 and GAD67, change their behaviour in people with chronic pain due to shifts in DNA methylation. For example, when GAD67, a gene involved in calming nerve signals, is switched off by these changes, it can lead to an increase in pain sensitivity [124]. Similarly, changes to how DNA is packaged (the acetylation of histones) can lead to increased inflammation in the spinal cord, which in turn makes pain feel worse and harder to control [125]. Furthermore, viruses are being used as delivery tools to bring healing genes directly into the nervous system. One promising example uses HSV (herpes simplex virus) vectors to carry anti-inflammatory molecules like IL-10 into inflamed nerves, and it was shown to reduce nerve pain in animal models without the side effects of pain medications [28]. Another technique involves silencing the NaV1.7 gene, which plays a big role in sending pain signals to the brain. When this gene is turned off using a method called LATER (Long-Acting Targeted Epigenetic Repression), pain signals are blocked without affecting other nerve functions [126]. By targeting the biological processes behind chronic pain, these methods offer good prospects for long-term relief that is beyond temporary fixes [127].

Traditional pain medications, such as opioids and NSAIDs, often affect the whole body, which unfortunately means they can cause a range of side effects, like drowsiness, nausea, constipation, and even problems with thinking and memory [50]. These systemic effects are worse for patients on long-term pain medications and can eventually lead to a reduction in quality of life and dependence and/ or tolerance over time. Gene and epigenetic therapies are different in that, instead of flooding the entire system, they are designed to target specific tissues or nerves involved in pain signalling. For example, viral vectors can deliver therapeutic genes directly to pain-sensing neurons in the DRG, which are key areas known for processing pain [117]. This targeted delivery helps reduce unwanted effects in other parts of the body. Similarly, epigenetic treatments can target specific genes or regulatory mechanisms that are associated with chronic pain. By adjusting how those genes are expressed, without altering the DNA itself, this way chronic pain can be treated without the widespread effects seen with traditional drugs [17]. This targeted/localised treatment makes these therapies safer and more tolerable for patients over time.

Challenges and limitations

The promise of gene therapy and epigenetic modulation in the treatment of chronic pain has opened a new frontier in pain medicine, offering a potential future where opioid dependency is no longer the cornerstone of management. However, as with all transformative innovations in medicine, this paradigm shift is not without significant challenges and limitations. The transition from theoretical promise to clinical reality remains fraught with technical, ethical, financial, and scientific complexities that demand scrutiny.

One of the most pressing technical barriers is the delivery of gene therapies and epigenetic modulators to specific pain-processing sites. Unlike systemic medications, these therapies often require targeted administration to DRG, spinal cord neurons, or specific cortical areas involved in pain perception [22]. Viral vectors such as AAVs have been employed to introduce therapeutic genes into neurons, but their ability to selectively target pain-relevant neuronal populations while avoiding off-target effects remains limited [128]. Moreover, the blood-brain barrier (BBB) poses a formidable obstacle to central nervous system (CNS) targeting, necessitating invasive delivery routes like intrathecal or intraparenchymal injections, which carry procedural risks [129].

Beyond physical delivery, achieving appropriate gene expression is another challenge. Overexpression or insufficient expression of introduced genes may result in ineffective treatment or adverse effects, particularly if gene products affect essential cellular functions [130]. Additionally, the transient or uncontrolled expression of epigenetic enzymes, such as HDACis or DNMTis, may alter non-targeted gene networks, potentially disrupting homeostatic gene regulation [17]. Thus, finely tuned regulation of these therapeutic interventions remains a key hurdle in clinical translation.

The ethical landscape surrounding gene and epigenetic therapies in chronic pain is equally complex. Altering the genome or its expression in neurons involved in pain modulation raises fundamental questions about long-term identity, consent, and potential off-target neuropsychiatric effects [131]. These concerns are amplified by the enduring nature of genetic interventions, which could persist beyond the therapeutic need or affect future neural plasticity in unpredictable ways [132]. Furthermore, ethical frameworks must consider the vulnerability of chronic pain patients, who may be more susceptible to consenting to experimental procedures due to desperation for relief, creating potential for coercion or therapeutic misconception [133].

Safety concerns also persist, particularly in light of immunogenicity and toxicity associated with viral vectors. While AAV vectors are generally considered safe, immune responses to the viral capsid or transgene product can lead to inflammation or neutralisation of the therapy, diminishing its efficacy and safety [134]. Non-viral delivery systems, such as nanoparticles or liposomes, may offer alternative platforms with reduced immunogenicity, but these too carry their own toxicity profiles and face challenges in bio-distribution and cellular uptake [135,136]. Additionally, epigenetic therapies must navigate the risk of oncogenesis, as the modulation of chromatin structure and DNA methylation can inadvertently activate proto-oncogenes or silence tumour suppressor genes [21].

The prohibitive cost of developing and administering gene and epigenetic therapies poses another critical limitation. Current gene therapy treatments for other conditions can exceed hundreds of thousands to millions of dollars per patient [137]. The infrastructure required for manufacturing, quality control, and clinical administration further escalates expenses, rendering these treatments inaccessible to most healthcare systems globally [138]. Insurance coverage and reimbursement remain significant hurdles, particularly in countries without universal healthcare systems. For chronic pain management, which affects millions worldwide, equitable access would necessitate substantial health policy reforms and subsidisation strategies.

Accessibility issues are compounded by the scarcity of specialised centres capable of administering these advanced therapies. Training of clinicians, establishment of regulatory pathways, and creation of standardised protocols are in their infancy, limiting the scalability of these interventions [139]. Moreover, the socioeconomic and racial disparities that plague current pain management systems may persist or even be exacerbated if advanced gene and epigenetic therapies are only available in high-resource settings [140]. Ensuring global accessibility will require international collaboration and a commitment to distributive justice in healthcare innovation.

Finally, the scientific community continues to grapple with the need for more robust and replicable data to support the widespread adoption of gene and epigenetic therapies in pain medicine. Many current studies are limited to animal models or small-scale clinical trials, with varying degrees of translational success [141]. The heterogeneity of chronic pain conditions complicates trial design, endpoint selection, and the generalizability of findings. Furthermore, long-term outcomes remain largely unexplored, with insufficient data on the durability of response, late-onset adverse effects, and optimal re-dosing intervals [142].

To address these gaps, ongoing research is exploring next-generation gene editing technologies such as CRISPR/Cas9 systems, which allow for more precise genomic alterations, and novel epigenetic modulators with improved specificity and reversibility [143]. Additionally, initiatives focused on developing pain biomarkers may facilitate patient stratification and more personalised therapeutic approaches, thereby enhancing trial efficiency and therapeutic efficacy.

Future directions

The ongoing opioid crisis has highlighted the urgent need for innovative strategies in chronic pain management. In response, researchers and clinicians are turning to gene therapy and epigenetic modulation as potentially transformative approaches. As these techniques evolve, their integration into clinical practice hinges on future directions in scientific advancement, technological refinement, ethical alignment, and equitable access. With a growing body of preclinical evidence and the emergence of early-phase human trials, future progress depends on overcoming existing hurdles while seizing opportunities presented by precision medicine and global collaboration.

The advent of CRISPR-Cas9 and other gene-editing tools has revolutionised biomedical research, enabling precise manipulation of specific genes associated with pain pathways. In animal models, targeted disruption of genes involved in nociceptive signalling, such as SCN9A (which encodes the voltage-gated sodium channel Nav1.7), has shown promise in abolishing pain perception without significant side effects [33]. The precision and efficiency of CRISPR, along with novel delivery vectors like AAVs and lipid nanoparticles, have expanded the potential to deliver gene therapies directly to sensory neurons or DRG [144]. Future directions will likely include refining these vectors for tissue-specific targeting, minimising off-target effects, and developing inducible systems that allow temporal control of gene expression [145].

Alongside gene editing, epigenetic modulation offers a complementary and potentially reversible strategy. Epigenetic marks such as DNA methylation, histone acetylation, and non-coding RNA expression can influence gene expression patterns involved in chronic pain states. For instance, persistent pain is associated with hypomethylation of pro-nociceptive genes and altered histone modification patterns in spinal cord neurons [17]. Drugs targeting HDACs and DNMTs are under investigation for their ability to restore epigenetic homeostasis in chronic pain [146]. Looking ahead, the development of highly specific epigenetic modulators that act only on pain-relevant genes will be crucial. Integration of single-cell sequencing and epigenomic mapping techniques will likely enhance the identification of key molecular targets and improve therapeutic specificity [147].

Future pain management may benefit from integrating these emerging therapies with existing pharmacologic and non-pharmacologic strategies to achieve a more comprehensive and individualised approach. While gene therapy and epigenetic tools provide the potential for disease modification or even reversal, they may be most effective when used alongside current modalities such as physical therapy, cognitive-behavioural therapy, and non-opioid medications [4]. For example, combining gene therapy that downregulates inflammatory mediators with physiotherapy may enhance recovery in patients with neuropathic pain. Similarly, epigenetic drugs might be used to sensitise patients to behavioural interventions by modulating pain memory circuits [148].

This multidisciplinary model aligns with the principles of precision medicine, which aims to tailor treatment based on individual genetic, environmental, and lifestyle factors. Advances in genomics and bioinformatics now make it possible to stratify patients based on genetic variants linked to pain perception and opioid response, enabling customisation of therapies [114]. For instance, patients with rare SCN9A mutations might be candidates for gene therapy targeting Nav1.7, while others may benefit from epigenetic modulators that correct aberrant gene expression in pain circuits [149]. Artificial intelligence and machine learning models may further facilitate the interpretation of large datasets to predict therapeutic responses and optimise treatment regimens [150].

The promise of these therapies, however, raises important questions about global accessibility and equity. Historically, the implementation of cutting-edge treatments has often been restricted to high-income countries, exacerbating existing disparities in pain management [151]. Future initiatives must prioritise infrastructure development, education, and cost-reduction strategies to ensure equitable access. The democratisation of gene editing tools through open-source platforms and international research collaborations may accelerate the translation of these therapies in low- and middle-income countries [152]. Public-private partnerships and global health frameworks could play a critical role in subsidising costs and facilitating clinical trials across diverse populations [153].

The safety and ethical implications of modifying the human genome for pain relief also warrant ongoing scrutiny. While somatic gene therapy does not alter the germline and is generally considered ethically acceptable, the irreversible nature of some interventions demands robust informed consent and long-term follow-up [154]. Regulatory frameworks must evolve to address these complexities, balancing innovation with patient safety. Future research will need to establish clear guidelines for risk-benefit assessment, especially in vulnerable populations or when therapies are applied prophylactically in high-risk individuals [155].

Emerging technologies such as optogenetics and chemogenetics may further refine the precision of gene therapy in chronic pain. These tools allow the activation or silencing of specific neuronal populations using light or designer drugs, offering a non-permanent and reversible means of neuromodulation [156]. When combined with gene editing or epigenetic interventions, such approaches may enable dynamic, real-time control of pain signalling. Research is also exploring RNA-based therapeutics, including siRNAs and antisense oligonucleotides (ASOs), which can transiently suppress pain-related genes without altering the genome [157]. Such developments may broaden the therapeutic arsenal and offer safer alternatives for patient populations where permanent genomic interventions are not advisable.

As the field matures, robust clinical trials will be essential to validate safety, efficacy, and long-term outcomes. To date, most studies have been limited to animal models or early-phase trials. Large, randomised controlled trials in diverse populations will be needed to understand variable responses, rare side effects, and the durability of therapeutic effects [158]. Additionally, the establishment of global registries and real-world evidence platforms can facilitate post-market surveillance and continuous learning.

## Conclusions

Gene therapy and epigenetic modulation together represent a paradigm shift in the understanding and management of chronic pain. By addressing the molecular and genetic underpinnings of pain perception, these approaches move beyond symptomatic control toward true disease modification. The ability to modulate neuronal excitability, inflammatory pathways, and maladaptive gene expression offers a pathway to long-lasting, personalized analgesia without reliance on opioids. However, realizing this vision requires overcoming significant translational challenges, including optimizing delivery systems, ensuring long-term safety, addressing ethical considerations, and improving accessibility. Collaborative, multidisciplinary research will be vital to bridge laboratory innovation with clinical application. As the boundaries between genetics, neuroscience, and pain medicine continue to blur, gene therapy and epigenetic modulation hold the potential to transform the landscape of chronic pain management, ushering in an era where sustained relief and functional recovery are achieved without the burden of opioid dependence.
